# Transcriptome Analysis of Tomato Leaves Reveals Candidate Genes Responsive to *Tomato Brown Rugose Fruit Virus* Infection

**DOI:** 10.3390/ijms25074012

**Published:** 2024-04-04

**Authors:** Donghai Wang, Mangle Chen, Jiejun Peng, Hongying Zheng, Yuwen Lu, Guanwei Wu, Jian Wu, Junmin Li, Jianping Chen, Fei Yan, Shaofei Rao

**Affiliations:** State Key Laboratory for Managing Biotic and Chemical Threats to the Quality and Safety of Agro-Products, Key Laboratory of Biotechnology in Plant Protection of Ministry of Agriculture and Zhejiang Province, Institute of Plant Virology, Ningbo University, Ningbo 315211, China; wangdonghai0539@163.com (D.W.); zhenghongying@nbu.edu.cn (H.Z.); zjlijunmin@126.com (J.L.);

**Keywords:** *tomato brown rugose fruit virus*, tomato, transcriptome analysis

## Abstract

*Tomato brown rugose fruit virus* (ToBRFV) is a newly-emerging tobamovirus which was first reported on tomatoes in Israel and Jordan, and which has now spread rapidly in Asia, Europe, North America, and Africa. ToBRFV can overcome the resistance to other tobamoviruses conferred by tomato *Tm-1*, *Tm-2*, and *Tm-2*^2^ genes, and it has seriously affected global crop production. The rapid and comprehensive transcription reprogramming of host plant cells is the key to resisting virus attack, but there have been no studies of the transcriptome changes induced by ToBRFV in tomatoes. Here, we made a comparative transcriptome analysis between tomato leaves infected with ToBRFV for 21 days and those mock-inoculated as controls. A total of 522 differentially expressed genes were identified after ToBRFV infection, of which 270 were up-regulated and 252 were down-regulated. Functional analysis showed that DEGs were involved in biological processes such as response to wounding, response to stress, protein folding, and defense response. Ten DEGs were selected and verified by qRT-PCR, confirming the reliability of the high-throughput sequencing data. These results provide candidate genes or signal pathways for the response of tomato leaves to ToBRFV infection.

## 1. Introduction

Tomatoes (*Solanum lycopersicum*) are an important vegetable crop produced all over the world which are constantly attacked by various pests and pathogens. Viruses are important pathogens that can reduce the yield, damage the quality and marketability of fruit, and cause huge economic losses [1,2,3,4]. *Tomato brown rugose fruit virus* (ToBRFV) belongs to the genus *Tobamovirus*, the genus in the family *Virgaviridae* with the largest number of species [5]. Four viruses in the genus can infect tomatoes. *Tobacco mosaic virus* (TMV) and *tomato mosaic virus* (ToMV) are the most widespread and long-known [6], while *tomato mottle mosaic virus* (ToMMV) was first found in Mexico in 2013 and ToBRFV was isolated from greenhouse tomatoes in Jordan in 2015 [7,8]. ToBRFV has spread rapidly and has now been reported from at least 35 countries across four continents (Asia, Europe, North America, and Africa) [9,10].

ToBRFV can infect more than 40 species of plants from four families, including the experimental hosts *Nicotiana glutinosa*, *N. tabacum*, and *Chenopodium quinoa* [11], but natural infections have only been reported on tomatoes and peppers [8,12]. Like other tobamoviruses, it has a single-stranded sense RNA molecule of about 6400 nucleotides, encoding four proteins [11]. There are two replication-related proteins: p126 and p183. P183 is encoded by readthrough at an amber stop codon UAG of the *p126* gene. The other two proteins are a ~30 kDa movement protein (MP) and the ~17.5 kDa coat protein (CP). P126 and p183 are directly translated from genomic RNA, while MP and CP are translated from subgenomic RNA. MP is necessary for virus cell-to-cell movement, while CP is involved in virus particle assembly and virus long-distance movement [9,10,13].

The symptoms caused by ToBRFV infection in tomatoes include yellowing and mosaic between the leaf veins; deformation and necrosis of young leaves and sepals; and the discoloration, deformation, marbling, and necrosis of young fruits. The severity of symptoms varies depending on the cultivar, growth stage, and culture conditions [8,10,11]. ToBRFV is mainly transmitted by mechanical contact, but it can also be transmitted over a long distance by contaminated seeds or fruits, like many other tobamoviruses [14,15,16]. Transmission between plants can occur through direct contact with infected plants, or from contact with diseased sap on farm tools, clothes, gloves, packaging boxes, etc. [9,17,18]. Levitzky et al. found that ToBRFV can also be spread through bumblebee pollination [19].

In tomatoes, three genes, *Tm-1*, *Tm-2*, and *Tm-2*^2^, confer resistance to TMV and ToMV, although some TMV and ToMV isolates can overcome this resistance [20]. However, tomato cultivars carrying *Tm-1*, *Tm-2*, and *Tm-2*^2^ genes are susceptible to ToBRFV isolates, and the incidence rate is close to 100% in some areas [8,11]. In recent years, naturally occurring ToBRFV-resistant and tolerant germplasms have been identified in tomato lines, tomato genotypes, and wild tomatoes [9].

Plants resist virus attack by rapid and comprehensive transcription reprogramming in infected cells. Previous studies have analyzed the response of plants to some tobamoviruses at the transcriptome level. For example, TMV-responsive genes encode many functional proteins, including transcription factors, antioxidant proteins, metabolic enzymes, and transporters [21]. Kalapos et al. compared the transcriptome changes induced by two different tobamoviruses in pepper leaves containing the *L3* resistance gene. Inoculation with *Obuda pepper virus* (ObPV) caused a hypersensitive response (incompatible interaction) and induced strong transcriptome changes, while inoculation with *pepper mild mottle virus* (PMMoV) led to systemic leaf infection without obvious symptoms (compatible interaction) and fewer transcriptome changes [22]. Jiao et al. analyzed the transcriptome changes in pepper leaves after inoculation with PMMoV and found that the expression levels of several autophagy-related genes were significantly increased after infection. Subsequent experiments found that autophagy played a positive regulatory role in the process of plant anti-PMMoV infection [23]. Li et al. and Sun et al. analyzed the transcriptome of watermelon fruits and leaves infected with *cucumber green mottle mosaic virus* (CGMMV) and identified many candidate genes and pathways involved in the response to the virus [24,25]. However, the gene expression of tomato leaves in response to ToBRFV has not been reported so far. Here, we used RNA sequencing technology to analyze the response of tomato leaves to ToBRFV and investigated the changes of gene expression between healthy leaves and virus-infected leaves. Our work has identified many candidate genes that can be used for further analysis of the ToBRFV–tomato interaction, providing in-depth insights for analyzing the mechanism by which tomatoes respond to ToBRFV infection.

## 2. Results

### 2.1. Phenotypic Confirmation of Tomato Line Inoculated with ToBRFV

**Figure 1:**
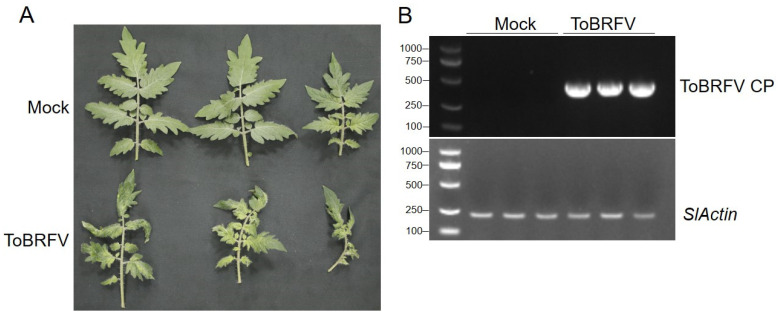
Symptoms on tomato leaves infected by ToBRFV. (**A**) Typical symptoms 21 days after ToBRFV inoculation. (**B**) Confirmation of the accumulation of ToBRFV in tomato leaves by PCR using ToBRFV CP-specific primers.

Tomato (Moneymaker) seeds were first tested by RT-PCR to ensure that they were not contaminated by ToBRFV. Seedlings with four leaves, 15 days after planting the seed, were inoculated by rubbing the leaves with sap from ToBRFV-infected plants, while the control plants were treated with phosphate buffer saline. After a further 21 days, the plants inoculated with ToBRFV showed symptoms such as leaf narrowing, shrinking, bubbling, and growth stagnation, whereas the control group developed normally (Figure 1A). RT-PCR analysis confirmed infection by ToBRFV (Figure 1B). Three biological samples each from the inoculated and control groups were sampled for high-throughput RNA sequencing by Novogene Bioinformatics Technology Co., Ltd. (Beijing, China).

### 2.2. Overview of RNA-Seq Data

The sequencing of the six samples produced a total of 40.43 G data, including 269,571,180 original reads (Table 1), which were enough for gene expression analysis. After eliminating low-quality bases, short reads, and adaptor sequences, 262,077,002 clean reads were obtained and the data efficiency was 97.22% (Table 1). Through HISAT2 (2.0.5) software, 251,503,263 reads were matched to the reference genome of the tomato, accounting for 95.97%. The Q20 ratio is higher than 97% and the Q30 ratio is higher than 93% (Table 1).

### 2.3. Analysis of Differentially Expressed Genes Caused by ToBRFV Infection

**Figure 2:**
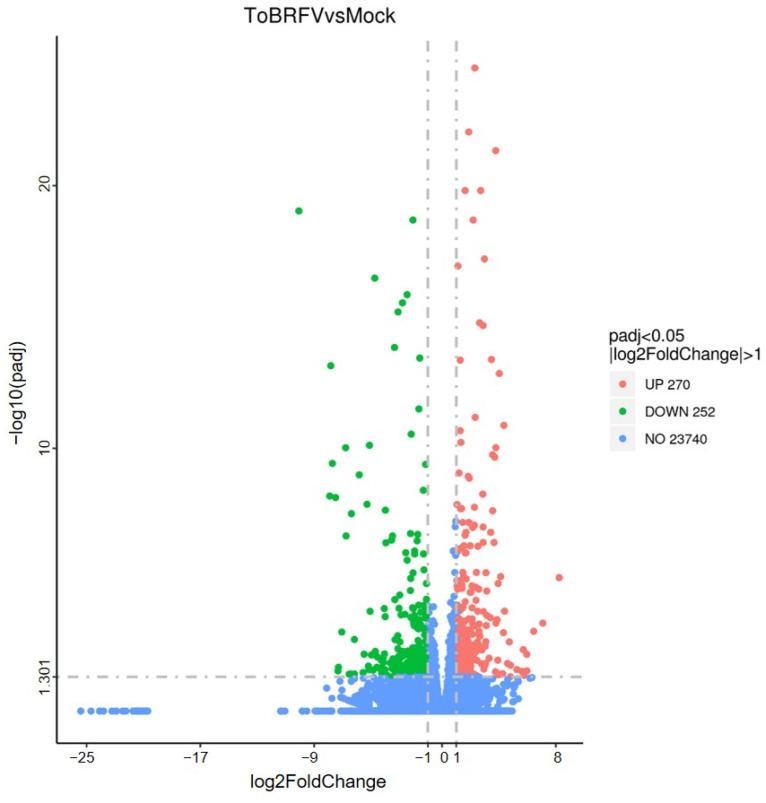
Volcanic diagram of the number of differentially expressed genes between tomato leaves of 21-day infected group and control group. Red dots represent up-regulated genes and green dots represent down-regulated genes.

Based on the FPKM (fragments per kilobase of transcript sequence per millions of base pairs sequenced) values of all genes in each sample, the correlation coefficients of samples within and between sample groups were calculated. It was found that the squared Pearson correlation coefficient between all biologically repeated samples was greater than 0.8, which indicated that the experimental operation was reproducible and the results obtained by subsequent differential gene analysis were reliable. Genes with an estimated absolute log2 fold change (log2FC) > 1 or <1 in sequence counts between libraries and with an FDR (false discovery rate) < 0.05 were considered to be significantly differentially expressed. In total, there were 522 differentially expressed genes (DEGs) between the ToBRFV-infected group and the control group, of which 270 were up-regulated and 252 were down-regulated (Figure 2; Table S1).

### 2.4. Gene Ontology (GO) and KEGG Analysis of DEGs

**Figure 3:**
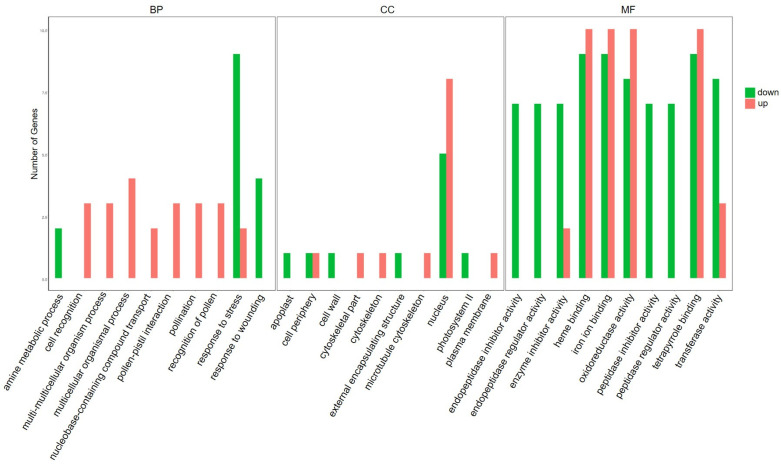
Gene ontology (GO) analyses of DEGs responsive to ToBRFV infection in tomato plants. GO analysis showing the GO terms in the categories of cellular component, molecular function, and biological process (FDR < 0.05). Red bars represent up-regulated genes and green bars represent down-regulated genes.

In order to classify the functions of DEGs, we used GO and KEGG pathway annotation to analyze the gene function enrichment. On the basis of GO classification, DEGs are divided into three categories: molecular function, cellular component, and biological process. In the biological processes category, there were significantly up-regulated genes in cell recognition, nucleobase-containing compound transport, response to stress, and other biological processes. In the category of cellular components, there were significantly up-regulated genes in the cell periphery, cytoskeleton, nucleus, and plasma membrane. In the category of molecular functions, significantly up-regulated genes included enzyme inhibitor activity, heme binding, iron–ion binding, oxidoreductase activity, and other functions (Figure 3). Significantly down-regulated genes in biological processes included the amine metabolic process, response to stress, response to wounding, etc. In the category of cellular components, apoplast, external encapsulating structure, nucleus, photosystem II, and other components were also significantly down-regulated, while in the molecular functions category, significantly down-regulated genes included endopeptidase inhibitor activity, endopeptidase regulator activity, and oxidoreductase activity (Figure 3).

**Figure 4:**
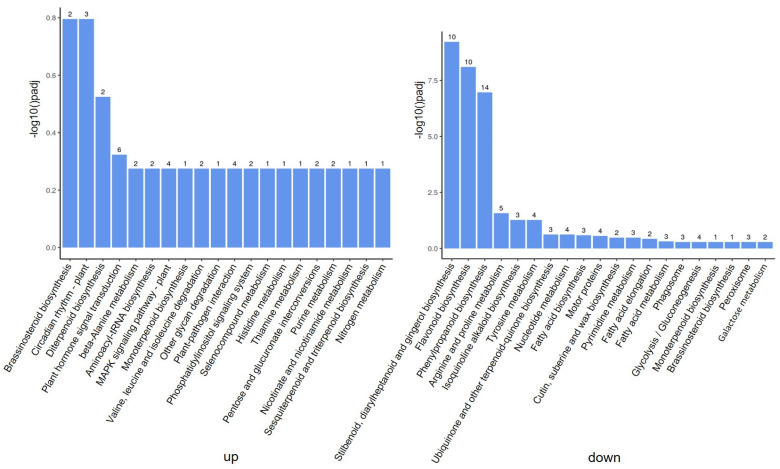
Kyoto Encyclopedia of Genes and Genomes (KEGG) analyses of DEGs responsive to ToBRFV infection in tomato plants. On the left are up-regulated genes and on the right are down-regulated genes. The number above the column indicates the number of differentially expressed genes in the enrichment process.

In order to further understand the molecular and biological functions of the DEGs, we mapped all differentially expressed unigenes into the KEGG database and compared the results with the whole transcriptome background. A total of 76 pathways were identified by pathway enrichment analysis. The genes up-regulated by ToBRFV infection occurred particularly in pathways such as brassinosteroid biosynthesis, circadian rhythm (plant), diterpenoid biosynthesis, and plant hormone signal transduction (Figure 4). The down-regulated genes were particularly involved in pathways such as stilbenoid, diarylheptanoid and gingerol biosynthesis, flavonoid biosynthesis, phenylpropanoid biosynthesis, arginine, and proline metabolism (Figure 4).

In addition, we used the STRING protein interaction database to predict the interaction network of differentially expressed genes. There are 15 node proteins and a total of 18 interaction proteins in the network constructed by up-regulated genes. Protein number 101256908 (*Solyc01g005300*), which encodes *Solanum lycopersicum* adagio protein 3, is the most predicted interacting protein (Figure S1, Tables S2 and S3). In the network constructed by down-regulated genes, 23 node proteins and 26 interacting proteins were predicted. The protein with the most interactions was predicted by protein number 101265455 (*Solyc07g053820*), which encodes the tomato mitotic checkpoint serine/threonine protein kinase BUB1 (Figure S1, Tables S2 and S3). In general, the expression levels of these genes and the number of proteins with which they interact are directly proportional to their importance. Therefore, the functions of these nodal proteins require further investigation.

### 2.5. qRT-PCR Confirmation of DEGs

**Figure 5:**
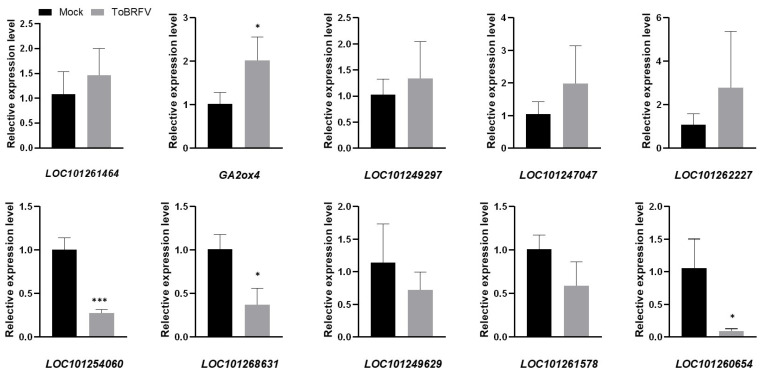
Validation of RNA-seq results. Ten differentially expressed genes were selected based on RNA-seq results, and their expression changes were analyzed by RT-qPCR with gene-specific primers. The obtained RT-qPCR data were normalized by *Slactin* expression level as the average value ± SD relative to the control inoculation. The asterisk indicates a statistically significant difference between the tomato plants inoculated with ToBRFV and the control. (* *p* < 0.05, *** *p* < 0.01).

RNA-Seq reveals the expression profiles of thousands of genes. In order to verify the results of RNA-Seq, we selected five DEGs from the up-regulated and down-regulated genes, respectively, and designed specific quantitative RT-PCR primers (Table 2). These genes were involved in basic plant metabolism, hormone signal transduction, gene expression regulation, etc. Among the quantitative PCR results from the up-regulated genes, *GA2ox4* was consistent with the transcriptome data, and *LOC101261464*, *LOC101249297*, *LOC101247047*, and *LOC101262227* were also induced by ToBRFV, although to a slightly lesser extent than that in the transcriptome data (Figure 5). Among the down-regulated genes, the quantitative results of *LOC101254060*, *LOC101268631*, and *LOC101260654* were consistent with the transcriptome data. The expressions of *LOC101249629* and *LOC101261578* were also significantly inhibited by ToBRFV, but not to such a great extent as in the transcriptome data (Figure 5). Overall, the results of qRT-PCR are similar to those from high-throughput sequencing, indicating that the results of the transcriptome analysis are reliable.

## 3. Discussion

ToBRFV has spread rapidly in countries around the world since it was first discovered, probably because of the global tomato seed and fruit trade [10]. The market value of tomatoes or peppers infected by ToBRFV is greatly diminished, so it is particularly important to explore the interaction between ToBRFV and its plant hosts. High-throughput RNA sequencing technology has been widely used to study the transcriptome changes of plants infected with different pathogens and has proved to be a reliable method to identify the host plant components involved in virus resistance when combined with gene function analysis. For example, Jiao et al. found that many autophagy-related genes (ATGs) were up-regulated by analyzing the transcriptome data of peppers infected by PMMoV. Confocal microscope observation showed that PMMoV could form a double-membrane autophagy structure after infecting plants. In addition, autophagy inhibitors could significantly increase the accumulation of virus RNA in plants, indicating that autophagy regulates the resistance of plants to PMMoV [23]. Xu et al. identified a number of potentially important DEGs by analyzing the transcriptome data of tobacco treated with Fe. After the functional analysis of these genes, it was found that genes such as *NbWRKY2* and *NbFAD3* negatively regulated PVY infection, while *NbCat-6A* positively regulated PVY infection [26]. Geng et al. revealed the dynamic changes of *N. benthamiana* at the transcriptome level by comparing the gene expression changes induced by wild-type and attenuated *tobacco vein banding mosaic virus* (TVBMV) with RNA-Seq technology, and found that wild-type and mutant TVBMV had different effects on the RNA interference and auxin signal transduction pathways [27].

Transcription factors (TFs) are composed of DNA-binding domains that interact with cis-regulatory elements of their target genes and protein interaction domains that promote oligomerization between transcription factors and other regulatory factors [28]. TF family members play important roles in plant transcriptional regulation. It has been previously reported that after pepper leaves were infected by ObPV, another tobamovirus, AP2/ERF; heat shock transcription factors; and NAC, WRKY, and ZAT family transcription factors were significantly up-regulated, while bHLH and TCP family transcription factors were significantly inhibited [22]. After CGMMV infects watermelon leaves, transcription factors of the MYB, NAC, zinc finger, bHLH, bZIP, WRKY, MADS box, WD-40, ERF, GRAS, and SBP-box families were significantly induced or inhibited [25]. In our study, 41 differentially expressed TFs were identified in tomato leaves infected with ToBRFV, of which 28 were up-regulated and 14 were down-regulated. These well-known TF families include ERF(5), NAC(5), bHLH(4), MYB(3), WRKY(3), HSF(3), etc. Previous studies have shown that there are six transcription factor families related to plant defense mechanisms: AP2/ERF, bHLH, bZIP, MYB, NAC, and WRKY [29]. In particular, some members of ERF, WRKY, and NAC transcription factors have been reported to be involved in antiviral defense responses in plants [22,25]. Our transcriptome results show that ToBRFV, like other tobamoviruses, can widely regulate the expression of transcription factors, and the role of these transcription factors in tomato resistance to ToBRFV deserves further study.

In our ToBRFV-induced transcriptome data, we found that MAPKKK (mitogen-activated protein kinase kinase kinase), WRKY transcription factors, Ca^2+^ binding protein, and other genes involved in the plant pattern-triggered immunity (PTI) pathway were significantly induced, indicating that virus infection can also activate the expression of PTI-related genes. Receptor-like kinases (RLKs) are a very important gene superfamily in plants, containing about 600 members in *Arabidopsis thaliana* and about 1000 members in rice [30]. They are widely involved in various biological processes in plants, such as growth, development, biotic and abiotic stress response, etc. [31,32]. Sun et al. reported that 26 differentially expressed RLK were identified in the transcriptome data of watermelon leaves infected by CGMMV, of which 16 were up-regulated and 10 were down-regulated [25]. In our transcriptome data, it was also found that dozens of RLK members were induced or inhibited in different degrees. There were 16 up-regulated RLKs (including leucine-rich-repeat RLKs, lectin RLKs, proline-rich RLKs, etc.) and 1 down-regulated RLK (LRR RLK), which indicated that RLKs were also widely involved in the tomato’s response to ToBRFV infection. The specific functions of these RLKs in response to ToBRFV need to be further studied.

Our results demonstrate the response of plants to ToBRFV at the transcriptome level for the first time, laying a foundation for studying the interaction between ToBRFV and its host plants and suggesting potential candidate genes that might be exploited to develop virus-resistant plants.

## 4. Materials and Methods

### 4.1. Plant Materials and ToBRFV Inoculation

Tomatoes (Moneymaker) were cultivated in a greenhouse at 26 °C with 16 h of light/8 h of darkness under an insect-proof net cover. After 15 days, plants were inoculated with ToBRFV by rubbing the leaves with infectious sap or with phosphate buffer saline solution as a control. The ToBRFV strain was isolated from Yuanmou County, Yunnan Province of China. Twenty-one days after inoculation with the virus, the systemic (upper, non-inoculated) leaves were sampled, the infection with ToBRFV was verified by RT-PCR, and suitable leaves were collected for high-throughput sequencing. Three biological replicates were obtained from each group.

### 4.2. Library Preparation for Transcriptome Sequencing

After extracting and quantifying the total RNA from the leaf sample, the plant mRNA was enriched by magnetic beads with Oligo (dT). Subsequently, fragmentation buffer was added to break the mRNA into short segments, and six-base random primers were used to synthesize single-strand cDNA using the mRNA as a template. Then, buffer, dNTPs, DNA polymerase I, and RNase H were added to synthesize double-strand cDNA, which was purified using AMPure XP beads. The purified double-stranded cDNA was first end-repaired, the A-tail was added and connected with a sequencing linker, and then the fragment size was selected by AMPure XP beads. Finally, PCR amplification was carried out, and the PCR products were purified by AMPure XP beads to obtain the final sequencing library. The qualified libraries were pooled and sequenced on Illumina platforms with the PE150 strategy in Novogene Bioinformatics Technology Co., Ltd. (Beijing, China).

### 4.3. Data Quality Control

For sequence quality control, the raw data (raw reads) in fastq format were first processed by internal scripts. In this step, clean data (clean reads) were obtained by deleting reads containing adapter, poly-N, or low-quality reads from the raw data. At the same time, the Q20, Q30, GC-content, and sequence repetition level of clean data were calculated. All downstream analysis was based on high-quality clean data.

### 4.4. Reads Mapping to the Reference Genome

The reference genome sequence and gene model annotation file of the tomato were downloaded from https://www.ncbi.nlm.nih.gov/datasets/genome/GCF_000188115.5/ (accessed on 31 March 2024) (Assembly accession: GCF_000188115.5). The reference genome was indexed by HISAT2 (v2.0.5) software, and the pair-ended clean read was matched to the reference genome by HISAT2 (v2.0.5) software.

### 4.5. Quantification of Gene Expression Level and Differential Expression Analysis

FeatureCounts (1.5.0-p3) was used to count the number of reads mapped to each gene. Then, based on the length of each gene and the number of reads mapped to the gene, the FPKM (fragments per kilobase of transcript sequence per millions of base pairs sequenced) was calculated. According to the FPKM values of all genes in each sample, the correlation coefficients of samples within and between groups were calculated, and the differences between groups and the duplication of samples within groups were displayed intuitively. Differential expression analysis between the inoculated and control groups (three replicates each) was performed using DESeq (v1.20.0) software. DESeq uses a model based on negative binomial distribution to provide a statistical method to determine the differential expression in digital gene expression data. The resulting *p*-values were adjusted using the Benjamini and Hochberg’s approach for controlling the false discovery rate. Genes that were determined by log2 (fold change) > 1 or log2 (fold change) < 1 with an adjusted *p*-value <= 0.05 found by DESeq2 were assigned as differentially expressed.

### 4.6. Gene Ontology (GO) and KEGG Enrichment Analysis

ClusterProfiler (3.8.1) was used for DEGs gene ontology (GO; http://www.geneontology.org) enrichment analysis. The GO term with a corrected *p* value < 0.05 was regarded as significantly enriched. Based on the KEGG database (http://www.genome.jp/kegg (accessed on 31 March 2024)), the statistical enrichment of differentially expressed genes in the KEGG pathway was tested by ClusterProfiler (3.8.1) software.

### 4.7. Validation of RNA-Seq Gene Expression Using qRT-PCR

In order to verify the gene expression level revealed by transcriptome data, the systemic leaves of tomatoes were collected for RNA extraction 21 days after ToBRFV (or mock) inoculation, with three biological replicates for each group. RNA was extracted by the TRIZOL method, and about 1 µg of total RNA was reverse-transcribed into cDNA by Transscript One-step gDNA Removal and cDNA Synthesis Supermix (Transgen Biotechnology, Beijing, China). cDNA was then used for real-time fluorescence quantitative PCR on a Roche LightCycler^®^480 Real-Time PCR instrument (Roche Diagnostics, Rotkreuz, Switzerland). The fold change of gene expression was estimated based on the expression of the housekeeping gene *Slactin* using the 2^−ΔΔCT^ method [33]. Cross-intron fluorescent quantitative PCR primers were designed according to the tomato genome information provided by the Solanaceae Genomics Network (https://solgenomics.net/), and the availability of primers was verified in advance. The primers’ sequence is listed in Table S4. The experiment was repeated three times independently.

## 5. Conclusions

In this study, we used high-throughput RNA-Seq technology for the first time to analyze the whole transcriptome of ToBRFV-infected tomato plants. The enrichment analysis of GO and KEGG pathways showed a series of different molecular changes at the global level caused by mock inoculation and ToBRFV inoculation, and showed the biological pathway of tomatoes infected by ToBRFV. The information provided in our study will be especially helpful to explore the pathogenesis of ToBRFV infection, study the molecular mechanism related to ToBRFV–tomato interaction, and identify the resistance genes in the future.

## Figures and Tables

**Table 1:** Summary of RNA-Seq data of tomato leaves.

Sample	Raw_Reads	Raw_Bases	Clean_Reads	Clean_Bases	Efftive (%)	Total_Map (%)	Unique_Map (%)	Q20 (%)	Q30 (%)	GC_pct
Mock-1	44142804	6.62 G	42813688	6.42 G	96.99	41303269 (96.47)	40580749 (94.78)	97.94	94.16	42.83
Mock-2	45319282	6.8 G	44126752	6.62 G	97.37	41552728 (94.17)	40210110 (91.12)	97.83	93.9	41.81
Mock-3	45272312	6.79 G	44359234	6.65 G	97.98	42787917 (96.46)	42075998 (94.85)	97.84	93.86	42.7
ToBRFV-1	46506486	6.98 G	45336348	6.8 G	97.48	43608492 (96.19)	42890628 (94.61)	97.74	93.55	42.5
ToBRFV-2	40698800	6.1 G	39633112	5.94 G	97.38	38129994 (96.21)	37542708 (94.73)	97.8	93.73	42.16
ToBRFV-3	47631496	7.14 G	45807868	6.87 G	96.17	44120863 (96.32)	43467843 (94.89)	97.99	94.27	41.92

**Table 2:** Ten candidate genes for quantitative PCR verification.

Gene_Name	Gene_Description	ck_1	ck_2	ck_3	21d_1	21d_2	21d_3	log2FoldChange	Regulation
*LOC101261464*	Solanum lycopersicum 26S proteasome non-ATPase regulatory subunit 2 homolog A	4.1752	7.539722	5.150759	87.30882	161.5379	93.72816	4.345529722	up
*GA2ox4*	Solanum lycopersicum gibberellin 2-oxidase	33.4016	31.23599	29.8744	222.4077	202.9852	195.267	2.715318985	up
*LOC101249297*	Solanum lycopersicum cytochrome P450 94A1-like	46.971	19.38786	81.38199	162.6701	461.2333	308.0879	2.655824219	up
*LOC101247047*	Solanum lycopersicum UDP-glycosyltransferase 74F2	154.4824	432.9955	631.483	1804.076	2948.067	1294.837	2.310442285	up
*LOC101262227*	Solanum lycopersicum benzyl alcohol O-benzoyltransferase	472.8414	610.7175	539.7995	2297.601	1775.855	3771.69	2.273012285	up
*LOC101254060*	Solanum lycopersicum fasciclin-like arabinogalactan protein 11	205.6286	208.958	201.9097	104.7706	55.26298	109.3495	−1.190321612	down
*LOC101268631*	Solanum lycopersicum probable leucine-rich repeat receptor-like protein kinase At5g49770	306.8772	336.0562	306.9852	128.6656	100.9612	174.4385	−1.230099258	down
*LOC101249629*	Solanum lycopersicum ribosomal protein eL27 pseudogene	79.3288	61.39488	47.38698	25.73313	28.69424	25.16775	−1.24276978	down
*LOC101261578*	Solanum lycopersicum salicylic acid-binding protein 2	829.821	961.8531	932.2873	439.3012	253.9971	429.5874	−1.27731831	down
*LOC101260654*	Solanum lycopersicum transcription factor MYB13-like	488.4984	1807.379	470.7794	47.79009	92.45921	135.3851	−3.326851689	down

## Data Availability

Sequencing data from this article were deposited at the CNGB Sequence Archive (CNSA) of China National GeneBank DataBase (CNGBdb) with accession number CNP0005400.

## Supplementary Materials

The following supporting information can be downloaded at: https://www.mdpi.com/article/10.3390/ijms25074012/s1.
