# Supplementary figures and images for: Transcriptome Analysis of Tomato Leaves Reveals Candidate Genes Responsive to Tomato Brown Rugose Fruit Virus Infection
Source: Int J Mol Sci. 2024 Apr 4;25(7):4012. doi: 10.3390/ijms25074012 (PMC11012278; doi:10.3390/ijms25074012)

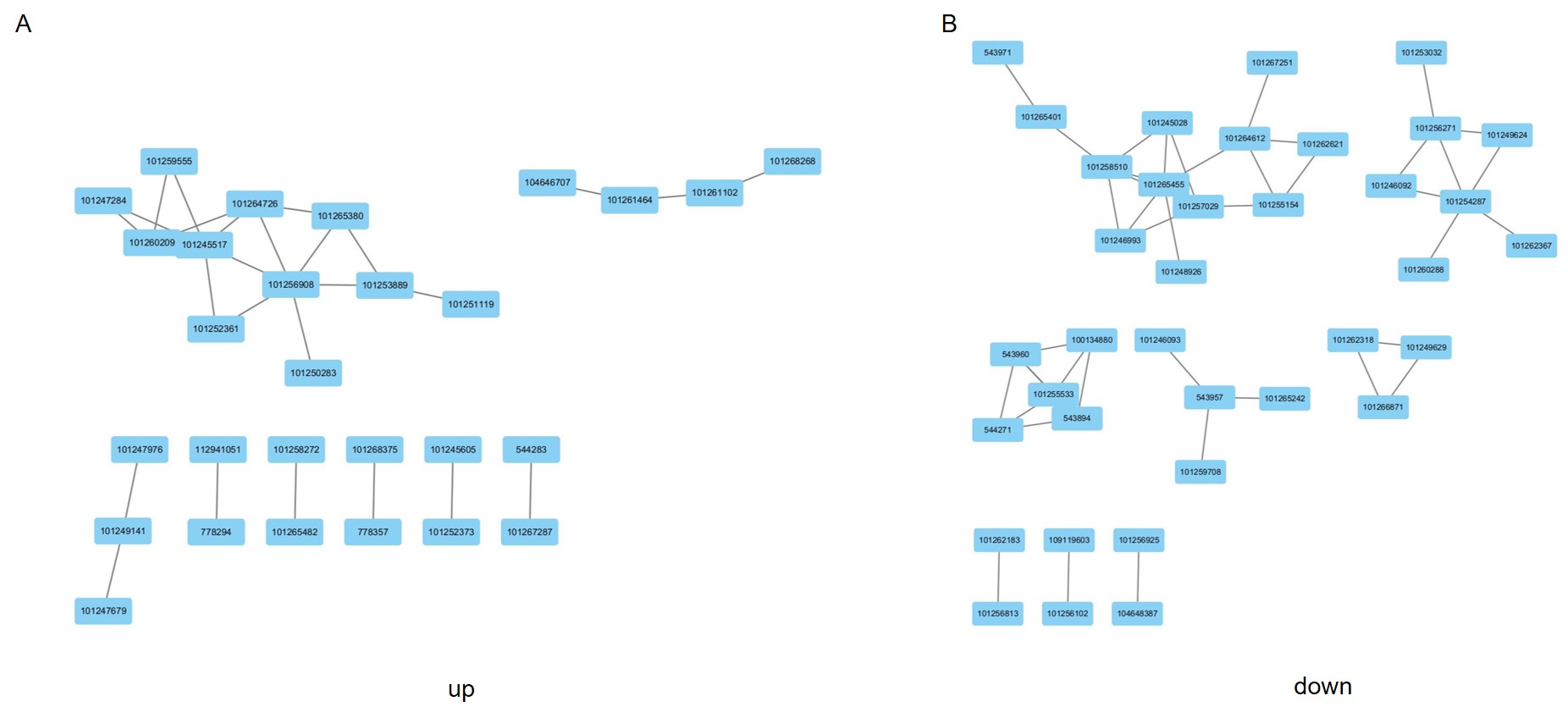

Supplement: Supplementary file 1 [file ijms-25-04012-s001.zip › Figure S1.jpg]
